# Is peer review useful in assessing research proposals in Indigenous health? A case study

**DOI:** 10.1186/1478-4505-7-2

**Published:** 2009-02-13

**Authors:** Jackie Street, Fran Baum, Ian PS Anderson

**Affiliations:** 1 Department of Public Health, Flinders University, Adelaide, South Australia, Australia; 2 Discipline of Public Health, The University of Adelaide, Adelaide, South Australia, Australia; 3 Onemda VicHealth Koori Health Unit, The University of Melbourne, Melbourne, Victoria, Australia

## Abstract

**Background:**

There has been considerable examination and critique of traditional (academic) peer review processes in quality assessment of grant applications. At the same time, the use of traditional research processes in Indigenous research has been questioned. Many grant funding organisations have changed the composition of their peer review panels to reflect these concerns but the question remains do these reforms go far enough? In this project we asked people working in areas associated with Aboriginal health research in a number of capacities, their views on the use of peer review in assessing Indigenous research proposals.

**Methods:**

In semi-structured interviews we asked 18 individuals associated with an Australian Indigenous research funding organisation to reflect on their experience with peer review in quality assessment of grant applications. We also invited input from a steering group drawn from a variety of organisations involved in Aboriginal research throughout Australia and directly consulted with three Aboriginal-controlled health organisations.

**Results:**

There was consensus amongst all participants that traditional academic peer review is inappropriate for quality assessment in Indigenous research. Many expressed the view that using a competitive grant review system in Aboriginal health was counterintuitive, since good research transfer is based on effective collaboration. The consensus within the group favoured a system which built research in a collaborative manner incorporating a variety of different stakeholders in the process. In this system, one-off peer review was still seen as valuable in the form of a "critical friend" who provided advice as to how to improve the research proposal.

**Conclusion:**

Peer review in the traditional mould should be recognised as inappropriate in Aboriginal research. Building research projects relevant to policy and practice in Indigenous health may require a shift to a new way of selecting, funding and conducting research.

## Background

The concept of peer review is changing. Traditional academic peer review is a system used for the assessment of quality of papers or grants whereby the work is critiqued by academic experts in the same field as the paper. It was widely adopted as a tool for quality assessment in the 1940s when systems for government support of scientific research were formalised and quickly gained a mantle of incontrovertibility [1]. More recent models are broader, incorporating technical and merit review, and often employing peers with skills in specific areas such as research transfer.

Criticism of traditional academic peer review focuses on: the potential for bias due to the power of elite researchers, conflicts of interest, contentious definitions of "excellence" and "peer", cost, lack of transparency, and its failure to detect fraud [1,2]. Recruitment of reviewers may be difficult since peer review provides few tangible benefits for reviewers and in Indigenous research there is the added burden of a small pool of researchers and an even smaller number of Aboriginal researchers. There is also little evidence to support the efficacy of peer review in improving the quality of research grant proposals [3]. Given these drawbacks, we suggest that continued strong support for peer review, as a system of quality assessment, may reflect the lack of viable alternatives.

Peer review does offer benefits to research-funding decision-makers in that it incorporates belief models integral to first world societies: the concepts of "healthy competition", "volunteerism", "free enterprise", and "democratic" devolution of decision-making to researchers. These beliefs are the product of a particular world-view, and are increasingly being challenged, not least in the Aboriginal research community. For example "healthy competition" on an unequal playing field may maintain inequity and "volunteerism" could be viewed as exploitation.

Underpinning the system is the belief that competent methodologically rigorous research will translate into improved policy and practice. Yet, although rigour is important, an overwhelming emphasis on excellence in research design and track record means that other factors, such as relevance for the community and potential for research transfer, may be neglected and these factors, in themselves, may compromise rigour.

Research grant assessment processes are important since they influence research priorities thereby shaping what evidence is available. The Global Forum for Health Research notes that most research conducted globally is not relevant to the health of poor people and very little is conducted on the broader determinants of health [4,5]. Others have pointed out that there is very little research which focuses on efficacy research (testing interventions in a controlled setting) or implementation research – the "how" of translating current research knowledge into practice within existing health and social systems [6,7].

The concept and practice of research in Indigenous communities in Australia and elsewhere is inevitably associated with the experience of colonisation [8] and research is still perceived by many Indigenous people as a tool for oppression and exploitation. One important strategy to address this issue has been increased recognition that Indigenous participation in research is essential. Kowal et al [9] attempted to clarify the meaning of 'participation' in the Indigenous context and pointed to the need for more debate in this area. We recognise that in some grant assessment processes, Indigenous researchers or Indigenous people working in end user sites are included as reviewers. However, the instances in which this is an uncontested practice are still relatively unusual – in the main, traditional academic peer review processes are considered normative.

This research project examines the role of traditional (academic) peer review in grant assessment processes for Indigenous health research and asks how these processes might be redesigned to better reflect community values and priorities and improve research transfer and health outcomes for Aboriginal people.

## Methods

We conducted semi-structured in-depth interviews by telephone or face-to-face with 18 individuals associated with an Aboriginal health research funding organisation, the Cooperative Research Centre for Aboriginal Health (CRCAH) [10]. The participants were chosen to provide a representative cross-section of researchers (potential and past grant applicants), staff, Board members, health service partners, academic partners, reviewers, and members of the research development group. Many served multiple roles in this regard. A steering committee of 12 persons drawn from Australian organisations within and outside the CRCAH, and including policy makers, was established. In addition, we directly consulted with three Aboriginal-controlled organisations. We have described in previous publications [11,12] the sampling frame for the research participants, the steering group protocol and a full list of the organisations represented.

A broad discussion document, underpinned by an extensive literature review, was distributed to all interviewees at least two weeks prior to their interview. This presented an overview of available evidence for the efficacy of peer review and a range of conflicting views on quality assessment processes in grant review. Interviewees were asked to reflect on their past experience of grant assessment process both within and outside the CRCAH. Interviews were recorded, transcribed and thematically coded with some themes directly arising from the interview schedule and others emerging from the interview findings or from recoding such as "tensions in the review process" [11,12]. Our data analysis was consistent with theoretical saturation on the major issues but given the contentious and innovative nature of the topic it is perhaps not surprising that maverick opinion on some facets continued to emerge throughout the study.

Feedback from all parties was sought for the final report arising from the project. Unless otherwise indicated the views expressed in the results are the majority views expressed by the participants. Interviewees were identified on the basis of current primary employment as academic (A) or community-based (C) with an additional identifying number.

A full description of the research method has been published previously [11] and a full report of the research findings has been described in a CRCAH discussion paper although the report focuses on the context of organisational change [12].

## Results

### Does peer review work in an Indigenous context?

There was universal consensus that traditional academic peer review is inappropriate for quality assessment in Indigenous research. Using competition to gauge excellence, in an arena where collaboration is essential for good research process and transfer, was seen as problematic. In addition, one participant commenting on the lack of evidence to support peer review, said it had always surprised him that the principal medical funding bodies purport to support a "commitment to evidence-based clinical practice to the nth degree and that there is this culture in the clinic that if you have got a marginal benefit on a randomised controlled trial, you have to ... change your practice" yet, by contrast, the "research is being funded by this absurd, highly subjective, highly manipulable, highly corruptible process" (A4).

Some saw the process as "more about supporting well-credentialed researchers who already have a history and record" than "encouraging and promoting research in Aboriginal communities" (C9). One participant suggested: "In reality, the critical mass of research people in this area are predominately non-Aboriginal and they have track records and in a competitive process they are always going to win" (C3). Track record, measured in terms of numbers of publications in peer-reviewed journals, is usually of paramount importance in academic assessment of grant applications but irrelevant in the Aboriginal community where the research may be carried out. One respondent questioned: "what is the credibility of someone with academic qualifications in an area where those carry no credibility whatsoever?" (C7) Several participants asked whether the emphasis on academic peer review was appropriate, with one suggesting: "traditionally too much research [has] focussed on the investigators' brilliance or not and it should not be about that, it should be about getting the best project which will get the best outcomes" (C8).

Some respondents saw peer review as a harsh process that undermined the ethos of trust which the CRCAH was trying to build. One participant described the process as "unproductive competition" causing "schisms" (A3) whereas others saw the problem in terms of being in a "competitive national research environment" in which community-driven projects "just don't fit" (C7).

Yet, many participants felt that academic peer review at some point in the grant review process is essential, principally to ward off allegations of "bias and nepotism" and to provide "transparency" and "rigour". One participant warned that peer review was needed to provide opportunities for external funding of CRCAH projects since "if our processes aren't peer review type processes, then our researchers won't be competitive" (A2).

### A paradigm shift?

Throughout the interviews considerable frustration was expressed about the gulf between the "two communities" [13] of academia and health services (denoted as the 'service industry' by our participants) and the difficulties in implementing change to reduce this gap. Both academic and health service personnel advocated a cultural change in the way research is done. One participant expressed this change as a paradigm shift: "to actually do research in a way that industry sees or perceives research, in how industry understands research, and how industry would actually know where it benefits within that, within service delivery for instance" (C6). This was deemed unlikely to occur as long as service industry personnel were peripheral to the decision-making process for grant funding. There was recognition that external pressures on the process were important, not the least being the financial pressures on academic institutions to meet external demands for high levels of publication and grant funding.

### Can we use review to develop better research?

In spite of the acknowledged difficulties, there was strong endorsement for building a system of review that would support a collaborative approach to research. In this context, a research-funding agency would act as a broker supporting the development of research projects that reflected community priorities. Support for this approach arose partly from what was seen as the fundamental difference between the goals of an Indigenous-controlled organisation and the mainstream funding bodies, as described by one participant: "We're not looking for excuses to not fund projects; we want to develop projects that will have relevant outcomes" (A5). Such a shift would involve moving from a system where the purpose of review is to sieve proposals to one where the objective is building effective research.

Similarly the role of a reviewer in a collaborative system would shift. In looking at how a review system might support research development some participants talked about the role of a "critical friend". In order to guide their choice, a group of people working up a project might ask: "whose wisdom would I really really value; who could really give me guidance because I trust them and I believe in them and I accept their critical perspectives?" (A1) Another participant suggested this input could be ongoing such that: "It's a continuing of the constructive suggestion process and so at a particular point your panel of constructive suggestions say, look I can't think of anything else to say" (A4). At this point the next question might be: "Do you proceed with the project?" (A4) There was some support for a review process external to the CRCAH at this stage.

### Who should assess quality in Indigenous research?

Agreement about who should be included as a "critical friend" in this system was less readily reached. Opinions ranged from an insistence that all assessors should have the skills and understanding of research and some degree of seniority, through to the view that senior personnel could be "too far up the rung to actually understand what is happening on the ground." (C6) More representation in the reviewer pool and on the review boards for "people with service delivery and grass roots" (C2) and "sharing the skill level and handing some of the skill level down" (C2) were seen as important by several participants. There was strong support for choosing peers with "a variety of different sorts of expertise to draw on" (A8) and recognition that the demands of a system of research development may conflict with local demands for community control. Such concern may have arisen from experience with academic peer review processes which appeared to undervalue local community needs. One researcher representing an important localised service-oriented project commented: "We thought we must be a priority, because we were trying to articulate an Indigenous community response which had been described by people...over a number of years" (C7).

It was also suggested that merit review might be separated from technical review. One participant noted: "some of the people who will look at it, won't necessarily be peers in the sense of researchers, but might be peers in the sense of people who understand Aboriginal health problems and understand Aboriginal communities and how they work" (A2). These reviewers might review the usefulness of the project whereas other reviewers might confine commentary to the methodology. The project group could draw on a range of expertise for "a full ranging discussion on a project in all its merit – in all its dimensions" (A4).

### Relevance

While the sample for this project was defined by the institutional reach of CRCAH, the organisations represented in the steering group and interviewee group covered a broad sweep across the Australian Aboriginal research area [11]. Therefore, with the caveat that there are particular peculiarities intrinsic to the CRCAH, such as the nature of the institutional partnerships [11], we would submit that the key findings may be applicable more broadly to the Aboriginal health sector and may find resonance with Indigenous health sectors in other countries. Beyond this, we would also contend that the insights provided by the research participants may also have relevance in constructing effective research transfer in other sectors, particularly in research carried out in the context of disadvantage and marginalisation.

## Discussion

This research describes some of the problems associated with the use of traditional peer review in an area where the existing power balance and control of the research process has been questioned [14]. In Indigenous research the use of traditional peer review can be a source of tension. Effective cross-collaboration of the health and research communities is important for effective transfer of research findings into policy and practice [13]. There is a danger that traditional peer review practice, since it disproportionately places power in the hands of selected academics, undermines attempts to bring the sectors together.

Traditional peer review tends to support mainstream, hypothesis-driven research (for example, see discussion in Wood [[1], p27]). This is a problem in an emerging field or innovative research where there may be only one "expert" and also in multidisciplinary or community-based research proposals, common in Indigenous research, where a single individual may not span the required expertise. Within Indigenous research the pool of available academic expertise is frequently limited: this may exacerbate a fundamental dilemma in peer review highlighted by Wood and Wessely [15] that there is a "trade-off between choosing reviewers who are indeed peers and the resulting increased chance of a conflict of interest."

Indigenous people, more generally, have, as a consequence of colonisation, been forced to operate within institutional structures that are not of their construction – which has created social marginalisation. It is perhaps inevitable that some Indigenous people remain hostile to participation in research processes. Nevertheless, our research also points to the willingness of many Indigenous people to engage in all aspects of the research process, including research assessment. Reforming research review to engage with Indigenous community perspectives has the potential to undermine this history of alienation and build a more collaborative research system (see our other publication, ref.[12], where this is discussed in more detail).

Jasanoff has called for "extended peer review" including stakeholder participation. [16] In Indigenous research there have been numerous calls for control by "Aboriginal communities in the design, execution and evaluation of research" [8,14,17-19]. Extended peer review might include the use of Indigenous community organisations and trained community peers in assessing research, for those factors which lie within the expertise of the community, but also might require that these organisations and peers be involved in the development of the research priorities and programs.

However, stakeholder review itself may present troubling issues. In the same way that peer review can represent a particular school of thought or cognitive cronyism [[1], p29], stakeholder review may bias the process in favour of a particular faction. Also, given the relatively small number of Aboriginal researchers and health workers, it is not clear from where stakeholders might be recruited.

If we wish to broaden the boundaries of peer review, the question arises as to what criteria might be used as a framework for review. Smith argues "the key to setting up any peer review system is knowing what you want your peer reviewers to do, and why" [20]. There is, however, "no consensus on its primary aim" [[21], p47]. There has been a shift in expectations from the belief that peer review would deliver research of superior methodological quality to a more complex set of parameters. A recent Cochrane systematic review looked at the effectiveness of grant peer-review processes on: importance, relevance, usefulness, soundness of methods, soundness of ethics, completeness and accuracy of funded research [3]. Many researchers would argue that traditional peer review can not address such a wide range of issues and perhaps should be confined to assessment of "intrinsic excellence" [[1], p89].

In addition, criteria for excellence may conflict with each other. Wood and Wessely [15] list seven key aspects which peer review is expected to meet: efficiency, effectiveness, accountability, responsiveness, rationality, fairness and validity but suggest that realisation of any one of these objectives may mitigate the realisation of another. They suggest that the "challenge for research funding bodies [is] to determine what constitutes a defensible/appropriate and workable balance" [15]. The system of collaborative research building described in this research and explored further in our other publications [11,12] may be one way of striking this balance.

## Conclusion

Underlying peer review is the assumption that the reviewer can assess potential for a grant proposal to translate into future productivity. Traditionally this productivity has been measured in terms of number of publications in peer-reviewed journals. However, our research supports the view that successful research must now include how the research is received by all parties involved in supporting healthy communities, including policy makers, practitioners and community organisations, and that research design and execution are only part of the equation. The role of stakeholder review must be to ensure that there is stakeholder commitment to the research and a determination to use the research. The value of including reviewers drawn from the service industry and policy arena must lie in the potential to improve the transfer of relevant research into policy and practice. As one participant commented:

"What we are trying to do, is not just do good research that will get published, we are trying to do good research that's going to lead to good outcomes" (A2).

## Abbreviations

CRCAH: Cooperative Research Centre for Aboriginal Health.

## Competing interests

FB and IA have served on the Research Development Group of the CRCAH. Currently IA is Research Director and FB is a Program Leader for the CRCAH. Apart from the funding provided for this project JS is entirely independent of the CRCAH.

## Authors' contributions

Research design by JS and FB with input from IA. JS managed the project and conducted the literature search and half of the interviews under the guidance of FB. The first draft of this paper was written by JS. FB and IA revised and edited this draft. All authors read and approved the final manuscript.
